# Supplementary material for: Bubble reachers and uncivil discourse in polarized online public sphere
Source: PLoS One. 2024 Jun 20;19(6):e0304564. doi: 10.1371/journal.pone.0304564 (PMC11189196; doi:10.1371/journal.pone.0304564)
Supplement: S1 Appendix — (PDF) [file pone.0304564.s001.pdf]

This appendix describes the alternative datasets applied in sensitivity analysis presented in S5 Appendix and S6 Appendix.

To address limitations related to the comparative datasets, particularly the exclusive collection of data for partisan accounts from the Facebook platform (for PARTISAN_pt and PARTISAN_REACHER_en), additional data was gathered. Specifically, we investigated to what extent the Facebook platform could influence the toxicity levels of comments to ensure a fair comparison between the comparative datasets. These datasets are summarized below.

FACEBOOK_NEUTRAL_en: obtained by selecting the top 3 Facebook pages by the number of comments from central leaning news media sources according to the All Sides Media Bias Report. Selected pages were: bbc, the_hill, abc_news.

FACEBOOK_PARTISAN_en: obtained by selecting the top 3 Facebook pages by the number of comments from left and top 3 from right-leaning news media sources according to the All Sides Media Bias Report. Selected pages were: cnn, msnbc, raw_story with left-leaning and fox_news, breitbart, the_blaze with right-leaning.

FACEBOOK_PERSON_en: obtained by selecting the top 3 Facebook pages from influential people (not news media) with the most comments in the dataset. Selected pages were: megyn_kelly, bill_mahar and rachel_maddow;

FACEBOOK_OTHER_en: obtained by randomly selecting 4 Facebook pages that weren't selected for FACEBOOK_NEUTRAL_en, FACEBOOK_PARTISAN_en or FACEBOOK_PERSON_en. Selected pages were: los_angeles_times, mother_jones, npr, and yahoo_news.

In addition to the alternative Facebook datasets, we also obtained comments from the Yahoo News site, which was labelled YAHOO_SITE_en. This dataset was used alongside the New York Times (NYT_SITE_pt) presented in the methodology section to conduct additional sensitivity analyses (described in S6 Appendix).

YAHOO_SITE_en: composed of $10,000$ random comments from Yahoo News website comments [123]. This dataset was selected to compare with comments obtained from yahoo_news Facebook page contained in FACEBOOK_OTHER_en to understand the influence of the Facebook platform by comparing the toxicity on comments made on Facebook and on the news website.
