# Supplementary material for: Bubble reachers and uncivil discourse in polarized online public sphere
Source: PLoS One. 2024 Jun 20;19(6):e0304564. doi: 10.1371/journal.pone.0304564 (PMC11189196; doi:10.1371/journal.pone.0304564)
Supplement: S2 Appendix — (PDF) [file pone.0304564.s002.pdf]

This appendix serves as a quick reference for datasets and associated sources. Tables 6, 7 and 8 present a summary of datasets and associated sources data for the Comparative, Reference and Alternative groups, respectively. Tables 9 and 10 provide summarized statistics for datasets and associated sources, respectively, including the initial comments count before and after pre-processing (Original and Final size columns, respectively), the percentage of comments that were removed after pre-processing (% column), and the mean, median and standard deviation values for the toxicity variable (Mean, Median and σ columns, respectively).

Table 6. Summary of datasets composing the Comparative group.

Dataset	Dataset summary	Source	Source summary
**NEUTRAL_REACHER_pt**	Comments captured from Brazilian news media websites during the 2018 presidential election representing the top three neutral bubble reaching domains in the Brazilian election setting: noticias.uol.com.br, g1.globo.com, and extra.globo.com [20].	extra.globo.com	Comments extracted on political topics during the 2018 Brazilian Presidential Elections from the news website extra.globo.com.
		g1.globo.com	Comments extracted on political topics during the 2018 Brazilian Presidential Elections from the news website g1.globo.com.
		noticias.uol.com.br	Comments extracted on political topics during the 2018 Brazilian Presidential Elections from the news website noticias.uol.com.br.
**NEUTRAL_REACHER_en**	Comments captured from Canadian news media websites during the 2019 federal elections representing the top three neutral bubble reachers in the Canadian election setting: cbc.ca, globalnews.ca, and theglobeandmail.com [20].	globalnews.ca	Comments extracted on political topics during the 2019 Canadian Federal Elections from the news website globalnews.ca.
		cbc.ca	Comments extracted on political topics during the 2019 Canadian Federal Elections from the news website cbc.ca.
		theglobeandmail.com	Comments extracted on political topics during the 2019 Canadian Federal Elections from the news website theglobeandmail.com.
**PARTISAN_pt**	Comments captured between 2012-01-01 and 2018-12-31 from Brazilian conservative Facebook pages: padrepaulo, flaviobolsonaro, CampanhadoArmamento, carvalho.olavo, DireitaConservadoraOficial, OPesadelodeQualquerPolitico2.0, and MisesBrasil [102].	CampanhadoArmamento	The Facebook page Campanha do Armamento is associated with the Instituto Defesa, a non-profit organization dedicated to advocating for and preserving the right to access firearms and self-defense in Brazil.
		MisesBrasil	The Facebook page Mises Brasil is associated with the Ludwig von Mises Brazil Institute (IMB), a think tank dedicated to producing and disseminating economic and social science studies that promote the principles of free markets and a free society.
		OPesadelodeQualquerPolitico2.0	The Facebook page O Pesadelo de Qualquer Politico 2.0 was a conservative page used to diffuse misinformation about politics. It is not available any more on the platform.
		Carvalho.olavo	This Facebook page is dedicated to disseminating the ideas and perspectives of Olavo de Carvalho, a Brazilian philosopher and political commentator known for his conservative and controversial views.
		Flaviobolsonaro	This Facebook page is associated with Flávio Bolsonaro, a right-wing Brazilian politician and senator, and serves as a platform for promoting his political agenda, sharing news and updates, and engaging with his supporters and followers.
		padrepaulo	This Facebook page is dedicated to promoting the teachings and activities of Father Paulo, a religious figure who shares spiritual guidance, discusses social issues, and engages with his followers through inspirational content and community outreach initiatives.
**PARTISAN_REACHER_en**	Comments captured from Facebook news pages representing the partisan accounts: bloomberg_politics, huffington_post, and ny_times [99].	huffington_post	The Facebook page Huffington Post is a platform for sharing news, opinion pieces, and engaging content covering a wide range of topics, including politics, culture, and current events.
		ny_times	The Facebook page New York Times provides comprehensive coverage of national and international news, investigative journalism, and in-depth analysis on various subjects, serving as a reliable source of information.
		bloomberg_politics	The Facebook page Bloomberg Politics offers insightful news and analysis on politics, policy, and global economic issues, providing readers with a deeper understanding of the intersection between business and politics.

Table 7. Summary of datasets composing the Reference group.

Dataset	Dataset summary
**UNCIVIL_en**	Highly uncivil comments in English obtained from an open dataset with human-labeled Wikipedia comments according to different categories of toxic behavior (`https://www.kaggle.com/competitions/jigsaw-toxic-comment-classification-challenge/data`).
**UNCIVIL_pt**	Highly uncivil comments in Brazilian Portuguese obtained from tweets manually annotated according to different toxicity categories [103].
**CIVIL_en**	More civil comments in English obtained through Reddit's public API from communities known for moderated discussions.
**CIVIL_pt**	More civil comments in Brazilian Portuguese obtained from a database of product reviews on a famous e-commerce website (B2W Digital) [104].

Table 8. Summary of datasets composing the Alternative group.

Dataset	Dataset summary	Source	Source summary
**FACEBOOK_NEUTRAL_en**	Top 3 Facebook pages [99] with comments from central leaning news media sources according to the All Sides Media Bias Report: bbc, abc_news, and the_hill.	bbc	The Facebook page BBC provides global news coverage across various topics including politics, current affairs, culture, and entertainment, with a focus on objective reporting and analysis.
		abc_news	The Facebook page ABC News delivers breaking news, features, and in-depth stories covering a wide range of topics, including politics, business, technology, and popular culture, with an emphasis on American perspectives.
		the_hill	The Facebook page The Hill offers political news, analysis, and opinion pieces covering US politics, Congress, the White House, and policy debates, catering to a wide audience including policymakers and political enthusiasts.
**FACEBOOK_PARTISAN_en**	Top 3 Facebook pages [99] with comments from left-leaning (cnn, msnbc, raw_story) and right-leaning (fox_news, breitbart, the_blaze) news media sources.	cnn	The Facebook page CNN delivers comprehensive news coverage on various topics including politics, business, world affairs, and entertainment, with a reputation for breaking news stories and in-depth reporting.
		msnbc	The Facebook page MSNBC offers liberal-leaning news, analysis, and opinion pieces on politics, social issues, and culture, catering to progressive viewers and providing coverage on a wide range of topics.
		raw_story	The Facebook page Raw Story features progressive news and investigative reporting, covering politics, social justice, science, and culture, with a focus on critical analysis and alternative perspectives.
		fox_news	The Facebook page Fox News provides conservative-leaning news and opinion pieces on current events, politics, business, and culture, with a focus on American perspectives and a strong following among conservative viewers.
		breitbart	The Facebook page Breitbart offers conservative news, opinion pieces, and analysis on politics, world events, culture, and technology, catering to a right-leaning audience and promoting conservative viewpoints.
		the_blaze	The Facebook page The Blaze provides conservative news, commentary, and analysis on politics, culture, and current events, offering a platform for conservative voices and catering to an audience seeking right-leaning perspectives.
**FACEBOOK_PERSON_en**	Top 3 Facebook pages [99] with comments from left-leaning (cnn, msnbc, raw_story) and right-leaning (fox_news, breitbart, the_blaze) news media sources.	person_rachel _maddow	Rachel Maddow's Facebook page features content from her television show, offering progressive commentary, analysis, and investigative reporting on politics, current events, and social issues from a liberal perspective.
		person_megyn_kelly	Megyn Kelly's Facebook page features content from her media ventures, offering news, interviews, and commentary on politics, culture, and current events, with a focus on delivering diverse perspectives and engaging discussions.
		person_bill_mahar	Bill Mahar's Facebook page features content from his shows, providing progressive commentary, satire, and political humor, with a focus on social and political issues, current events, and interviews with notable figures.
**FACEBOOK_OTHER_en**	Randomly selected 4 Facebook pages [99] not included in FACEBOOK_NEUTRAL_en, FACEBOOK_PARTISAN_en, or FACEBOOK_PERSON_en: los_angeles_times, mother_jones, npr, and yahoo_news.	npr	The Facebook page NPR delivers news, analysis, and storytelling on a wide range of topics including politics, culture, science, and arts, with a reputation for in-depth reporting and high-quality journalism.
		yahoo_news	The Facebook page Yahoo News offers a mix of news articles, videos, and features covering politics, current events, lifestyle, and entertainment, catering to a broad audience with diverse interests.
		los_angeles_times	The Facebook page Los Angeles Times provides comprehensive news coverage on local, national, and international stories, including politics, entertainment, sports, and culture, with a focus on the Los Angeles area.
		mother_jones	The Facebook page Mother Jones offers progressive news, investigative reporting, and commentary on politics, social justice, and environmental issues, with a reputation for in-depth reporting and inquisitive journalism.
**NYT_SITE_en**	Comments from the New York Times website (https://www.kaggle.com/datasets/aashita/nyt-comments).	Politics	5,000 random comments from politics related sections.
		Non-politics	5,000 random comments from non-politics related sections.
**YAHOO_SITE_en**	10,000 random comments from Yahoo News website [123].	–	–
**G1_SITE_pt**	Comments obtained from the g1.globo.com domain during different contexts other than the 2018 Brazilian presidential elections [105].	Economia	This column covers the latest updates and analyses on financial markets, business news, economic policies, and trends affecting Brazil and the global economy.
		Bem Estar	This column provides information and tips on nutrition, fitness, mental health, and lifestyle choices for a balanced and healthy life.
		Educação	This column explores educational topics, including school policies, educational reforms, advancements in teaching methods, and initiatives aimed at improving the education system in Brazil.
		CIÊNCIA E SAÚDE	This column covers groundbreaking research, medical advancements, public health issues, and scientific discoveries in various fields to keep readers informed about the latest developments.
		Agro	This column delves into the agricultural sector, discussing farming practices, rural policies, market trends, innovations in agriculture, and the impact of agriculture on the economy and society.
		Auto Esporte	Focusing on the automobile industry, this column provides news, reviews, and insights on cars, motorcycles, and related technologies, keeping readers updated on the latest trends in the automotive world.
		Eleições 2020	This column covered the 2020 elections in Brazil.
		Política	Covering domestic and international politics, providing insights into the political landscape in Brazil and beyond.

Table 9. Datasets size before and after pre-processing and statistics.

	Size			Toxicity		
Dataset	Original	Final	%	Mean	Median	σ
NEUTRAL_REACHER_pt	128,898	122,836	95.30	0.44	0.42	0.27
PARTISAN_pt	30,000	24,741	82.47	0.32	0.20	0.30
NEUTRAL_REACHER_en	115,779	113,114	97.70	0.17	0.10	0.17
PARTISAN_REACHER_en	31,075	28,070	90.33	0.24	0.16	0.23
CIVIL_pt	5,000	4,989	99.78	0.12	0.06	0.15
CIVIL_en	5,000	4,976	99.52	0.19	0.11	0.19
UNCIVIL_pt	5,000	4,718	94.36	0.87	0.90	0.14
UNCIVIL_en	5,000	4,927	98.54	0.95	0.96	0.06
FACEBOOK_NEUTRAL_en	60,155	57,839	96.15	0.23	0.14	0.23
FACEBOOK_PARTISAN_en	121,928	118,910	97.52	0.27	0.20	0.24
FACEBOOK_PERSON_en	70,717	70,290	99.40	0.30	0.24	0.24
FACEBOOK_OTHER_en	60,337	56,404	93.48	0.22	0.13	0.23
G1_SITE_pt	342,405	328,026	95.80	0.47	0.50	0.26
NYT_SITE_en	256,507	255,012	99.42	0.19	0.15	0.16
YAHOO_SITE_en	10,000	9,026	90.26	0.17	0.08	0.20

Table 10. Sources size before and after pre-processing and statistics.

		Size			Toxicity		
Dataset	Source	Original	Final	%	Mean	Median	σ
NEUTRAL_REACHER_pt	extra.globo.com	51	49	96.08	0.59	0.67	0.25
	noticias.uol.com.br	69,814	67,779	97.09	0.45	0.47	0.25
	g1.globo.com	59,033	55,057	93.26	0.42	0.40	0.27
PARTISAN_pt	CampanhadoArmamento	5,000	4,199	83.98	0.36	0.28	0.29
	MisesBrasil	5,000	3,508	70.16	0.29	0.18	0.27
	OPesadelodeQualquerPolitico2.0	5,000	4,306	86.12	0.46	0.45	0.32
	carvalho.olavo	5,000	4,218	84.36	0.32	0.20	0.30
	flaviobolsonaro	5,000	4,307	86.14	0.30	0.16	0.28
	padrepaulo	5,000	4,203	84.06	0.16	0.07	0.20
NEUTRAL_REACHER_en	theglobeandmail.com	13,328	13,144	98.62	0.17	0.10	0.17
	cbc.ca	100,358	97,932	97.58	0.17	0.10	0.16
	globalnews.ca	2,093	2,038	97.37	0.28	0.16	0.25
PARTISAN_REACHER_en	bloomberg_politics	961	888	92.40	0.23	0.15	0.22
	ny_times	15,754	14,169	89.94	0.22	0.15	0.21
	huffington_post	14,360	13,013	90.62	0.26	0.17	0.24
FACEBOOK_NEUTRAL_en	the_hill	20,490	20,070	97.95	0.28	0.20	0.25
	abc_news	16,598	15,663	94.37	0.22	0.11	0.22
	bbc	23,067	22,106	95.83	0.20	0.11	0.19
FACEBOOK_PARTISAN_en	the_blaze	15,112	14,714	97.37	0.25	0.18	0.23
	raw_story	20,076	19,703	98.14	0.35	0.31	0.27
	breitbart	20,218	19,175	94.84	0.28	0.18	0.27
	msnbc	21,207	20,917	98.63	0.27	0.21	0.22
	cnn	22,325	21,618	96.83	0.24	0.17	0.21
	fox_news	22,990	22,783	99.10	0.26	0.20	0.22
FACEBOOK_PERSON_en	person_rachel_maddow	24,123	24,092	99.87	0.32	0.28	0.22
	person_megyn_kelly	22,747	22,488	98.86	0.24	0.17	0.21
	person_bill_mahar	23,847	23,710	99.43	0.34	0.29	0.25
FACEBOOK_OTHER_en	los_angeles_times	11,465	10,234	89.26	0.24	0.16	0.24
	yahoo_news	14,421	13,668	94.78	0.23	0.16	0.21
	npr	16,959	15,396	90.78	0.15	0.07	0.18
	mother_jones	17,492	17,106	97.79	0.27	0.20	0.24
G1_SITE_pt	Economia	34,518	33,437	96.87	0.40	0.36	0.26
	Bem Estar	65,606	63,011	96.04	0.45	0.45	0.26
	Eleições 2020	725	702	96.83	0.39	0.34	0.26
	Educação	5,227	5,086	97.30	0.47	0.49	0.26
	CIÊNCIA E SAÚDE	2,492	2,410	96.71	0.38	0.34	0.27
	Agro	2,024	1,962	96.94	0.46	0.49	0.25
	Auto Esporte	1,747	1,683	96.34	0.30	0.22	0.26
	Política	230,066	219,735	95.51	0.49	0.54	0.25
NYT_SITE_en	Politics	57,934	57,502	99.25	0.22	0.18	0.17
	Non-politics	198,573	197,510	99.46	0.19	0.14	0.15
YAHOO_SITE_en	–	10,000	9,026	90.26	0.17	0.08	0.19
