# Supplementary material for: Bubble reachers and uncivil discourse in polarized online public sphere
Source: PLoS One. 2024 Jun 20;19(6):e0304564. doi: 10.1371/journal.pone.0304564 (PMC11189196; doi:10.1371/journal.pone.0304564)
Supplement: S3 Appendix — (PDF) [file pone.0304564.s003.pdf]

The pre-processing step was performed to maintain maximum integrity in the datasets presented in Section Materials and Methods, only removing noisy instances that could negatively impact performance during toxicity identification. Escape sequences (such as line breaks), Markdown tags, and links have been removed. Sentences repeated in more than one comment, such as “Reply to @” and “REPORT COMMENT,” which are clearly not part of the construction of a comment, were also identified and removed.

Since online comments tend to be noisy, usually containing spelling errors, it was necessary to apply an autocorrection step. For this task, an algorithm called *SymSpell* (`https://symspellpy.readthedocs.io`) was applied, which depends on the creation of a dictionary with the correct number of words that will be used to replace those whose spelling does not conform to the dictionary standard. Thus, a dictionary was created for each dataset, including the words that appeared at least 5 times in the respective dataset, given that more frequent tokens tend to be the correct version. In contrast, rare tokens tend to be spelling errors. Then, into this initial dictionary, we concatenated a standard dictionary of the language of each dataset, Brazilian Portuguese for datasets with _pt prefix and United States English for datasets with _en prefix. These dictionaries were obtained from the OpenOffice repository (`https://www.openoffice.org/lingucomponent/dictionary.html`). The resulting dictionary is then processed by *SymSpell*, which generates permutations of the words through the character deletion procedure, resulting in a dictionary of permutations with words that would potentially be misspellings. After this procedure, the comments' autocorrection was performed. In this step, *SymSpell* was configured to autocorrect only words with a maximum edit distance of 2 characters (the number of characters needed to turn a misspelled word into the correct word). It was observed that the increase to a maximum edit of 3 or more characters generated incorrect respellings. Short words with wrong spelling were more likely to be replaced by words with correct spelling but unrelated to the corrected word. It is important to mention that the autocorrection step does not eliminate the possibility of interference from adversarial attacks [124], but it can reduce their incidence.

Considering that even with the autocorrection procedure, the comments could still present noise from rare or unusual words, a pre-processing step was added to remove comments with many poorly recognized instances. For this, the comments were

processed by a tool widely applied in the literature to recognize linguistic, psychological, and social characteristics called LIWC [125]. This tool was chosen because it has good coverage for several dictionaries of different languages and does not require complex steps in the pre-processing of the text to be analyzed. For datasets in English, the official internal LIWC dictionary was used, and an unofficial dictionary [126](`http://143.107.183.175:21380/portlex/index.php/pt/projects/liwc`) for datasets in Brazilian Portuguese. After processing the datasets with LIWC, the "Dic" attribute was used to keep only comments with at least 50% of the words recognized by the respective LIWC dictionary – this attribute counts the percentage of words identified in the respective dictionary. Finally, considering that short comments can influence toxicity results, all comments containing less than 10 words were removed using LIWC's WC (Word Count) parameter, which counts the number of words identified in a text.
