# Supplementary material for: Bubble reachers and uncivil discourse in polarized online public sphere
Source: PLoS One. 2024 Jun 20;19(6):e0304564. doi: 10.1371/journal.pone.0304564 (PMC11189196; doi:10.1371/journal.pone.0304564)
Supplement: S4 Appendix — (PDF) [file pone.0304564.s004.pdf]

S4 Fig.

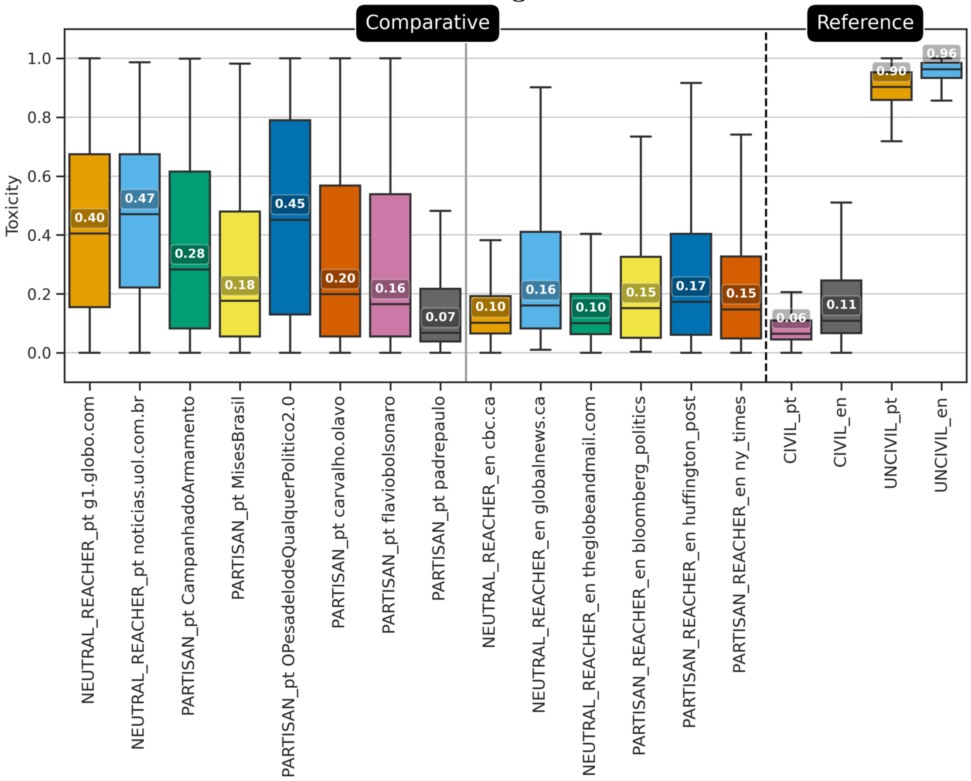

Fig . Box plots showing the distribution of toxicity scores grouped by source for the comparative and reference comments datasets.

Our central hypotheses concern whether neutral bubble reachers tend to reduce uncivil discourse. We found that they do in Canada but not in Brazil. To probe this result further, we examined specific accounts to understand if their distinctive histories and audiences might impact results, as well as whether the commenting systems and moderation policies they use would perhaps generate more heated discussions. For this purpose, we conducted the same analysis for the datasets in Incivility and neutral bubble reachers section. S4 Fig shows the toxicity score box plots grouped by source for this analysis.

This figure reveals valuable insights that were not possible with Fig 2. Noticias.uol.com.br and g1.globo.com showed a similar toxicity level for the Brazilian neutral bubble reachers. This suggests that the toxicity level was likely not a direct product of those platforms, but more probably related to the general political situation of the country.

Regarding the Canadian neutral bubble reachers, both theglobeandmail.com and

cbc.ca exhibited very low toxicity scores, while, compared to them, globalnews.ca shows a 1.6 times higher toxicity median score. This finding is interesting for at least two reasons. For one, it suggests that if globalnews.ca had produced toxicity scores similar to the other Canadian news sources, the effect of neutral bubble reachers would have been even stronger. Second, it raises questions about why globalnews.ca differs from the others. There are a number of possible explanations for this difference. For example, the CBC is a government news source, and The Globe and Mail is one of Canada's oldest national news organizations with a strong reputation for being moderate. Global News, by contrast, is a newer source that grew out of talk radio, a legacy that might carry forward into it, hosting more lively and heated conversations. Another possibility concerns the commenting systems used by the sites. CBC requires users to sign into their site to comment and use a verified email address and does not permit pseudonyms. The Globe and Mail requires users to have a verified subscription to comment. By contrast, Global News' discussion board is automatically integrated with Facebook. This difference might account for the greater toxicity of Global News comments, in that Facebook conversations may tend to be more heated and less cautionary, and also are not confined to subscribers. This observation motivates additional sensitivity analysis of the impact of Facebook on toxicity levels presented on S5 Appendix and S6 Appendix.

To verify if the differences between toxicity scores observed in S4 Fig were statistically significant, we applied the tests explained in Section Methods for Hypothesis Investigation (the same one applied for datasets in Section Incivility and neutral bubble reachers, but changing the tested variables to be the Comparative group sources toxicity scores presented on Fig S4 Fig. These sources were subjected to a normal test applying D'Agostino K-squared test [111]. Table 11 presents these test results, showing that none of the datasets followed a normal distribution ($p < .001$). We also relied on Q-Q plots and histogram visualizations for each source, confirming this result (plots were not included for brevity). Considering that the data were not following a normal distribution, we applied the Kolmogorov-Smirnov Goodness of Fit test [112] pairwise between sources to verify whether the samples originate from the same distribution. Results for this test were included in a separate spreadsheet for consultation (`https://zenodo.org/records/10443022`), which shows that none of the Comparative sources originated from the same distribution ($p < .05$), except for the

case of the pair of sources ny_times and bloomberg_politics, which the test showed that were originated from the same distribution. Therefore, the observed differences in box plots are statistically significant, except for this specific case.

Table 11. D'Agostino K-squared (k^2) tests for Comparative sources.

Dataset	Source	k^2
NEUTRAL_REACHER_pt	g1.globo.com	982,481.04***
	noticias.uol.com.br	92,809.12***
PARTISAN_pt	flaviobolsonaro	559.56***
	MisesBrasil	409.62***
	OPesadelodeQualquerPolitico2.0	27,061.59***
	padrepaulo	1,299.81***
	carvalho.olavo	1,017.08***
	CampanhadoArmamento	2,932.33***
NEUTRAL_REACHER_en	cbc.ca	40,039.28***
	theglobeandmail.com	5,002.15***
	globalnews.ca	312.46***
PARTISAN_REACHER_en	huffington_post	1,626.50***
	ny_times	2,478.50***
	bloomberg_politics	174.65***

Note: k^2 represent the D'Agostino K-squared [111] statistic. All statistics are significant at $p < .001$ (***) level.
