# Supplementary material for: Bubble reachers and uncivil discourse in polarized online public sphere
Source: PLoS One. 2024 Jun 20;19(6):e0304564. doi: 10.1371/journal.pone.0304564 (PMC11189196; doi:10.1371/journal.pone.0304564)
Supplement: S5 Appendix — (PDF) [file pone.0304564.s005.pdf]

Overall, our analysis suggests that neutral bubble reachers have a variable effect on uncivil discourse. Some evidence suggests that in more highly polarized contexts, their performances of neutrality lose credibility, thereby eliciting greater toxicity. The strength of our results, however, relies on controlling for unaccounted sources of bias. One limitation in the analysis was that data on ideologically partisan accounts were exclusively collected from Facebook, raising the possibility that the results may be unique to the affordances of this platform.

We therefore compare the datasets with comments obtained from Facebook pages to identify whether Facebook accounts for different toxicity scores over and above bubble reaching. This is important for determining if the toxicity levels identified in the PARTISAN_pt and PARTISAN_REACHER_en datasets is potentially a product of partisan accounts, or if it is more likely a product of Facebook's platform own users' normal behaviour. In the first case, the toxicity scores should be different between the datasets, while in the second case, the toxicity scores should be similar.

S5 Fig show the toxicity box plot to compare comments on distinct Facebook pages. The vertical gray line in this figure separates the Brazilian Portuguese (left) from the English pages (right).

The differences between pages in both languages are clearly noticeable, suggesting that toxicity scores are not products of the Facebook platform alone. On the PARTISAN_pt dataset, for example, there are cases with relatively low toxicity, like padrepaulo's page, but also cases with the highest toxicity among other pages, like in OPesadelodeQualquerPolitico2.0 and Campanha do Armamento. When it comes to personal pages from famous personalities, like rachel_maddow (political news commentator) and bill_mahar (also a political news commentator, but in a satirical tone), the toxicity scores are slightly higher compared to pages from news media outlets on FACEBOOK_OTHER_en and PARTISAN_REACHER_en. It is worth noting that PARTISAN_REACHER_en sources, in general, had median values close to the sources in FACEBOOK_NEUTRAL_en, except for the the_hill Facebook page. Also, the sources in FACEBOOK_PARTISAN_en and FACEBOOK_PERSON_en have median toxicity scores higher than the sources in PARTISAN_REACHER_en and FB_CENTER_en, except for the the_hill Facebook page. Overall, variation in toxicity across these pages suggests that toxicity is not a constant feature of the Facebook platform, and the selection of

S5 Fig.

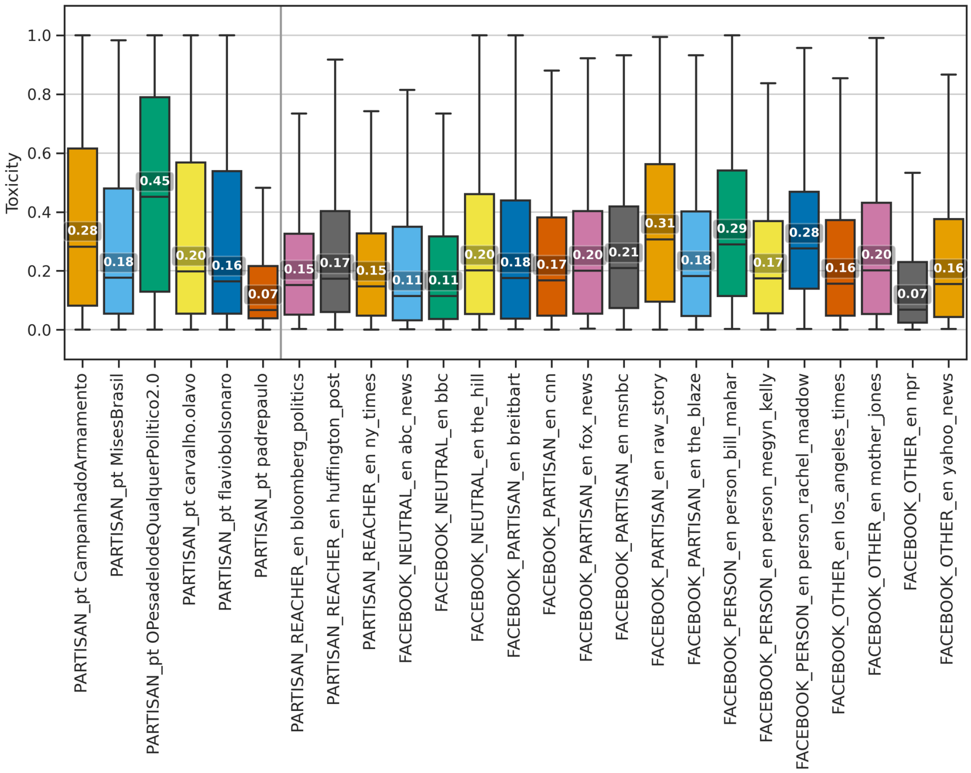

Fig . Box plots showing the distribution of toxicity scores for the Facebook Pages. The vertical gray line in this figure separates the Brazilian Portuguese (left) from the English pages (right).

sources from this platform should not systematically bias our earlier analysis.

To verify if the differences between toxicity scores observed in S5 Fig were statistically significant, we applied the tests explained in Section Methods for Hypothesis Investigation (the same one applied for datasets in Section Incivility and neutral bubble reachers, but changing the tested variables to be the Facebook sources toxicity scores presented in this figure). These Facebook sources were subjected to a normal test applying D'Agostino K-squared test [111]. Table 12 presents these test results, showing that none of the Facebook sources followed a normal distribution ($p < .001$). We also relied on Q-Q plots and histogram visualizations for each Facebook source, confirming this result (plots were not included for brevity). Considering that the data were not following a normal distribution, we applied the Kolmogorov-Smirnov Goodness of Fit test [112] pairwise between Facebook sources to verify whether the samples originate from the same distribution. Results for this test were included in a separate spreadsheet for consultation (`https://zenodo.org/records/10443022`), which shows that none of the Facebook sources originated from the same distribution ($p < .05$). Therefore, the observed differences in box plots are statistically significant.

Table 12. D'Agostino K-squared (k^2) tests for Facebook sources.

Dataset	Source	k^2
PARTISAN_pt	CampanhadoArmamento	2932.33***
	MisesBrasil	409.62***
	OPesadelodeQualquerPolitico2.0	27,061.59***
	carvalho.olavo	1,017.08***
	flaviobolsonaro	559.56***
	padrepaulo	1,299.81***
PARTISAN_REACHER_en	bloomberg_politics	174.65***
	huffington_post	1,626.50***
	ny_times	2,478.50***
FACEBOOK_NEUTRAL_en	abc_news	2,280.71***
	bbc	3,797.29***
	the_hill	2,072.24***
FACEBOOK_PARTISAN_en	breitbart	2,114.90***
	cnn	2,332.63***
	fox_news	2,048.13***
	msnbc	1,845.90***
	raw_story	3,887.21***
	the_blaze	1,464.03***
FACEBOOK_PERSON_en	person_bill_mahar	3,225.41***
	person_megyn_kelly	2,687.70***
	person_rachel_maddow	1,880.39***
FACEBOOK_OTHER_en	los_angeles_times	1,614.26***
	mother_jones	1,650.20***
	npr	4,354.89***
	yahoo_news	1,469.69***

Note: k^2 represent the D'Agostino K-squared [111] statistic. All statistics are significant at $p < .001$ (***) level.
