# Supplementary material for: Bubble reachers and uncivil discourse in polarized online public sphere
Source: PLoS One. 2024 Jun 20;19(6):e0304564. doi: 10.1371/journal.pone.0304564 (PMC11189196; doi:10.1371/journal.pone.0304564)
Supplement: S6 Appendix — (PDF) [file pone.0304564.s006.pdf]

To ensure that our previous results are not biased by platform selection, we consider comments made in each news media on their Facebook account or website. In the ideal case, we could compare comments on the same articles on different platforms, but this data was unavailable. Therefore, we compare toxicity scores for a set of articles on the news organization's own website to another set of articles on Facebook. We were able to make this analysis for Yahoo News and the New York Times. To achieve this, we sourced the comments from each of these media outlets based on their origin (website or Facebook page). Concerning Yahoo News, the website comments were sourced from the YAHOO_SITE_en dataset, while comments from its corresponding Facebook page were extracted from the yahoo_news page within the FACEBOOK_OTHER_en dataset. In the case of the New York Times, website comments were extracted from the NYT_SITE_en dataset, and comments from its Facebook page were extracted from the ny_times page within the PARTISAN_REACHER_en dataset.

A linear regression analysis was performed, considering toxicity scores as the dependent variable and the comment's origin (website or Facebook page) as an independent variable. The results revealed statistically significant values for Pearson's coefficients in both cases ($p < .001$), with $r = 0.144$ for Yahoo News and $r = 0.044$ for the New York Times. This result indicates that comments on Facebook had slightly higher toxicity scores than on the website for Yahoo News; in the case of the New York Times, there is no correlation. Thus, we have a suggestion that Facebook's influence on toxicity results is perhaps more minimal than one might expect. The fact that we have different articles of the same news media on different platforms could help explain this minimum influence observed for Yahoo News.

Although this new analysis helps in understanding the influence of the platform where the comments were made, it holds a limitation due to the unavailability of data for directly comparing comments on the same articles across different platforms. Consequently, the comparison made between toxicity levels on these news organizations' websites and their corresponding Facebook page might not fully capture the complete impact of platform-specific differences on toxicity. This is a topic worth exploring in future work.
