# Supplementary material for: Bubble reachers and uncivil discourse in polarized online public sphere
Source: PLoS One. 2024 Jun 20;19(6):e0304564. doi: 10.1371/journal.pone.0304564 (PMC11189196; doi:10.1371/journal.pone.0304564)
Supplement: Attachment — Submitted filename: Responses.pdf [file pone.0304564.s007.pdf]

Bubble reachers and toxic discourse in polarized online public sphere

Jordan Kobellarz, Milos Brocic, Daniel Silver and Thiago H. Silva

To the Editor and Reviewers.

We would like to thank you for reviewing our manuscript. We have revised the paper taking into account the Reviewers' remarks and suggestions, which were quite helpful. We believe that we have a much better version of the manuscript after incorporating those changes. Below, we present our responses to specific comments.

Responses

Reviewer #1

Comment 1: "1. There are two issues surrounding language and framing that I would recommend the authors reconsider:

a. The outcome variable is framed largely in terms of "toxic discourse", but I'm not persuaded that this is the most appropriate construct for approaching the questions at hand. In it's simplest terms, the issue at stake here is whether politically neutral brokers/bridges in a network lead to more civil, democratic deliberations or whether they contribute to incivility and hostile interpretations of content/information. It seems that "civility" and "democratic discourse" are more commonly accepted and clearly defined outcomes. It's not necessarily self-evident that "toxicity" is the antithesis of deliberation, and it's been argued in some contexts that effective democracy can be "rude". At a minimum, I would recommend that the authors be a bit more deliberate (though this can be brief) about connecting these concepts and addressing the value of democratic deliberation (regardless of its tone). Ideally, it would be more compelling if the findings could be discussed in the context of deliberation and its import."

b. The other area that I would recommend considering is the language around “bubble reachers”. This may be an artifact of different disciplinary groundings, but in network studies the more traditional terms would be “brokerage/brokers” and/or “network bridges”. This may create some confusion for diverse disciplinary audiences. At a minimum, I would recommend connecting/explaining this diverse terminology early in the manuscript. (There are some areas where the authors use the term “Brokerage”, but I’m not sure the terminology is ever clearly explained in an inter-disciplinary manner).”

>>> Thank you very much for helping us clarify our intentions regarding the language and framing.

In relation to **point a)**, we agree that ‘toxic discourse’ is not purely harmful, and that it is not necessarily at odds with deliberative democracy. We have now included a couple of sentences citing studies which make this point, and find that toxic discourse can bolster participation, and accompany reason giving:

> *“Toxic discourse is not necessarily incompatible with deliberative democracy, to be sure [12]. Heated debates naturally involve aggressive and conflictual discourse, and studies find that this can make discussions more engaging while still contains appealing to deliberative persuasion [13–15]. Be that as it may, toxic discourse is regarded as both symptomatic of polarization, and as a fuel that exacerbates it further [16]. Research finds that toxic discourse can elicit a feedback loop that provokes it in others, that it alienates more civil participants from platforms, and distorts perceptions of political life altogether [17, 18]. While it may not be inherently harmful to deliberative political talk, it is deemed problematic to the extent that it contributes to polarization and intolerance [12].”*

This information can be found on Page 5, Lines 103-112. While we stuck with the toxic discourse framing, we clarified now that the issue is more so its relation to polarization rather than deliberation.

In relation to **point b)**, we have provided an explanation of the association between the term "bubble reacher" and more widely recognized network theory concepts, such as "broker" and "brokerage." This association includes the incorporation of concepts derived from existing literature on weak ties (Granovetter, 1977) and structural holes (Burt, 1995). We have included the following paragraph in the section dedicated to related work:

> *“The term “bubble reachers” refers to users on social media platforms who distribute content that reaches users with diverse political opinions, possibly bridging ideological divides and*

facilitating cross-partisan conversations [9]. They play a role in countering the phenomenon of ”filter bubbles” [21] or ”echo chambers,” [22] where individuals are exposed more frequently to information and perspectives that reinforce their prior beliefs. To understand the concept of bubble reachers in a broader network science context, we can draw parallels with the terms “brokerage” and “brokers.” In network science, brokerage refers to the role played by individuals or entities that connect otherwise disconnected groups or individuals within a network [23]. They occupy positions that span structural holes [23], which are gaps between distinct clusters or communities within a network. By spanning the structural holes, brokers create weak ties [24], acting as intermediaries and facilitating the flow of information, resources, or relationships between different parts of the network. Bubble reachers can be seen as performing a form of brokerage between partisan groups in a polarized context. They bridge ideological gaps and act as connectors between users with diverse political opinions, similar to how brokers bridge gaps between different groups or clusters in a network.”

This information can be found on Page 6, Lines 126-143.

Comment 2. There are also a few additional areas where I would recommend briefly building out the literature in order better situate the findings across diverse disciplinary perspectives. I think that the authors’ findings will resonate deeply with those who study confirmation bias and hostile media effects. I would recommend leaning a bit more into the language of both literature bases (both to better contextualize the findings and to increase the study’s exposure across niche disciplines). There is also a bit more literature to consider re: the “echo chamber hypothesis” (such as recent work by Dubois and Blank, 2018) which suggests that the societal impacts of online filter bubbles may be overstated (though I would argue that there is still reason to be concerned about this phenomenon).

>>> Thank you for this suggestion. We agree that our analysis fits well the literature on confirmation bias and hostile media effects. This is especially the case with regards to how they intersect with populism. We have now incorporated literature from this subfield in our literature review on Page 7, Lines 178-181:

“Consistent with this, Schulz, Wirth and Müller [41] find that populist citizens subscribe to a “false consensus”, assuming that their opinions are overwhelmingly shared by others, but unfairly maligned by hostile and biased mainstream media.”

We have also included a citation to Dubois and Blank in our introduction, where we note limitations to the echo chamber argument (Page 2, Lines 25-26):

> *"However, recent research has largely popped the bubble on the ideological bubble perspective [11, 12]."*

Comment 3. There are some areas (particularly early in the manuscript) that need additional citations. Some examples include "Plato" (pg. 2), "These, to some scholars, evoked the forms of" (pg. 2), "A common response… has been to encourage exposure…" (pg. 2); "the claim to objectivity… provoke greater scorn as a result" (pg. 7), etc.

>>> We have now added additional citations where needed.

Comment 4. The authors note that data include comments related to "articles published during the electoral period in each country". This period should be clearly defined for each country, as should the context of the elections in question. The authors do an excellent job of framing each country's context as it relates to populist sentiment, but what about the specific context of these elections? Were they tightly contested elections? Were they "landslide" elections? Were there specific economic or social conditions that drove electoral debates? And do these factors affect the interpretation of findings in any way?

>>> Thank you for your comment. We have provided an explanation of each specific political situation during the 2018 Brazilian Presidential Elections and the 2019 Canadian Federal Elections as follows:

> *"For that, understanding the political context surrounding each election is fundamental for a comprehensive analysis. In the case of Brazil, during the election period, the polls[3] revealed a notable degree of polarization among voters. The electoral contest revolved around two primary candidates: Jair Messias Bolsonaro, who symbolized the potential for a 15-year departure from the ruling Workers Party (PT), and Fernando Haddad, who represented the continuity of PT's governance. This situation arose after a brief period during which a PT president elected was impeached and replaced by her vice-president. Ultimately, Bolsonaro emerged victorious with a majority of 55.13% of the valid votes, while Haddad secured 44.87% of the votes in his favor[4]. In Canada, Justin Trudeau represented the Liberals, which had previously held a parliamentary majority after unseating the Conservatives in 2015. In a close election, the Liberals won 157 (39.47%) seats in parliament, while the Conservatives, led by Andrew Scheer, won 121 (31.89%)[5]. The (left-wing) New Democratic Party continued*

to lose ground from its 2011 peak, especially in French Quebec, where the Liberals and the separatist Bloc Québécois subsequently gained ground. The 2019 election resulted in the Liberals forming a minority government, which have historically exhibited instability, since the prime minister relies on representatives of other parties to remain in power[6]. Despite Canada's multi-party political system, national politics has predominantly revolved around the traditional left-right ideological differences since at least the 1980s [77]."

This information can be found on Page 12, Lines 298-316.

Comment 5. Lastly, I did find the naming of data sets to be a bit hard to follow. The data sets are similarly named and I often found myself having to go back and forth to remind myself which was which. I wonder if the use of more conceptual terminology when referring to the data sets would be easier for readers. (i.e. Neutral Brazilian Sources, Partisan Brazilian Sources, etc.)

a. I would also recommend including a little more specific info about who these sources are (perhaps in the "Supporting Information").

>>> Thanks for the suggestion to improve the names used for the datasets. We did our best to try to identify names that were easier to interpret, using a more conceptual terminology, but this ended up making the text much more verbose and difficult to understand. For example, BIASED_pt would become "Partisan Brazilian Conservative Facebook Pages (pt)" and BIASED_en would become "Partisan Facebook News Pages (en)", which is much more difficult to relate one to another and repeat this naming convention across all text, tables and figures.

Considering that, we chose to keep the naming convention used previously and create an appendix on "Supporting information" section summarizing all datasets and respective sources, so that the reader can refer quickly while reading the text. This information can be found in the newly added S1 Appendix "Datasets and related sources summary".

Comment 6. Again, I think that the study makes a valuable contribution to the literature. My comments and recommendations are largely focused on clarifying some terminology, improving the readability of the manuscript, and better situating the findings across relevant disciplines.

>>> Thanks for the recommendations and interest in our work.

Reviewer #2

Comment 1. "This paper aims to compare levels of toxicity in comments using two case-studies, Canada and Brazil, to advance the idea that some media outlets and accounts may serve as 'bubble reachers' and breach into polarized networks. While the focus on a comparative case is welcome and so is the inclusion of the Brazilian case, as there is still little research focused on global south countries, the contributions of this specific project are not clear to me for both conceptual and methodological reasons. Conceptually, the idea of bubble reachers and the operationalization of these accounts seem to come from another study by some of the authors, and the current paper simply takes on accounts identified as bubble reachers and conducts an analysis of their comments, doing little to expand on, or demonstrate, the role of bubble reachers."

>>> Thanks for this comment. In reference to the past research referenced by the reviewer, we view the present article as taking a substantial step forward by analyzing the style of communication provoked by neutral bubble reachers. Past research has demonstrated that bubble reachers, despite their key structural position in online networks, do not necessarily lead users to break out of their ideological bubbles. While politically diverse users engage with bubble reachers' content, they disproportionately share content that aligns with their own partisan orientations. This raises the question that motivates the present research: do neutral bubble reachers perhaps serve a communicative function that goes beyond "popping the bubble" by convening discursive spaces that are less toxic than elsewhere? We found, however, that this is not always the case. Understanding this shift to the tone and style of discourse associated with neutral bubble reaching is the core advance of the present paper over past work. We tried to clarify our main contributions on bubble reachers' role by including the following content in the updated version of our manuscript:

In the Introduction:

> *"Addressing these issues is the central goal of this paper: we examine how ideologically neutral bubble reachers differ from ideologically partisan accounts in the level of toxic discourse they provoke, and explore how this varies in a context where populism is more and less prominent in political culture. We do that by analyzing the communication style provoked by neutral bubble reachers, ensuring a substantial step forward regarding past research. In this regard, we evaluate four hypotheses: 1) that more ideologically neutral bubble reachers*

should provoke less toxic comments relative to ideologically partisan accounts; 2) that toxic discourse will be more pronounced in contexts where political culture is marked by populism and polarization; that in these contexts 3) neutral bubble reachers will spark greater toxic discourse than their partisan counterparts and that 4) even non-political context will elicit toxic discourse. We explore these hypotheses by examining Brazil and Canada. While populism has deep roots in Brazil's political culture, Canadian political culture is comparatively moderate.

We find some support for our hypotheses: ideologically neutral bubble reachers support less toxic discourse in Canada, but the opposite relationship holds in Brazil. Here, even non-political content by ideologically neutral bubble reachers elicits considerable toxic discourse. Understanding this shift to the tone and style of discourse associated with neutral bubble reachers is the core advance of the present paper over past work."

This change can be found on Page 4, Lines 65-83.

And in the Discussion and Conclusion section:

"This study represents a significant advancement from previous research by analyzing the communication style provoked by neutral bubble reachers. The findings from this study could help researchers, platform designers, and policymakers seeking to promote healthier online interactions. Recognizing the contextual factors that impact the effectiveness of neutral bubble reachers can inform the development of strategies to mitigate toxicity and foster more constructive dialogue in online spaces. Moreover, understanding the interplay between polarization, populism, and the performance of mediating institutions can contribute to efforts to address the broader challenges associated with political polarization and the erosion of civility in public discourse."

This change can be found on Page 30, Lines 816-824.

Comment 2. Second, the study that drives the conceptualization and operationalization of bubble reacher accounts is based on Twitter data, not Facebook, and network features and associations across the two platforms are sufficiently different to warrant a validation of the concept, its ‘representative accounts’, and its role in a different network. That this may not be

possible due to data collection restrictions on Facebook is understandable—however, in that case, what can we really say about bubble reachers on Facebook and what is the validity of extending the observations made on Twitter to this platform considering such limitations?

>>> Thank you for raising this important point. While it is true that we operationalize our neutral bubble reachers by relying on findings from Kobellarz et al. 2022 which uses Twitter data, there are several reasons why we think the extrapolation to Facebook is valid. First, the neutral bubble reachers that emerged from the Twitter analysis were major legacy media outlets. That these outlets were central in reaching across ideological bubbles is consistent with other work likewise finding evidence that legacy outlets have the greatest audience overlap in consumption of digital news (see Mukerjee, Majó-Vázquez, and González-Bailón 2018). This makes us confident that the neutral bubble reachers identified using Twitter data should characterize these outlets more broadly, including on Facebook. Since the mechanisms we discuss are related to features of legacy media outlets (i.e. their appeals to civility, impartial mediation, and the challenge posed to them by populism), this should apply across platforms including Facebook.

Second, Facebook dataset allows increasing the information diversity on comments, extrapolating the exclusive use of Twitter as a source, which would make results skew to Twitter platform practices and political representativeness of users – *"Twitter users who write about politics tend to be male, to live in urban areas, and to have extreme ideological preferences."* (see Barbera and Rivero, 2014). Twitter platform demand discourses that are simple (given the character limit), inhibits reflexivity (also, given how easy it is to post something), and encourage uncivil discourse (given the impersonal characteristic of the platform) (see Ott, 2017). Also, previous work on polarization and hate speech relied heavily on data from Twitter alone, and in ours we extrapolate to other platforms to study the phenomenon more broadly.

We added a paragraph on the article explaining all these points to make more clear our rational about extrapolating Twitter data:

> *"It is important to consider the validity of using Facebook data, considering that neutral bubble reachers were identified from Twitter data [86]. First, the neutral bubble reachers that emerged from the Twitter analysis were major legacy media outlets. That these outlets were central in reaching across ideological bubbles is consistent with other work likewise finding evidence that legacy outlets have the greatest audience overlap in consumption of digital news [87]. This makes us confident that the neutral bubble reachers identified using Twitter data should characterize these outlets more broadly, including on Facebook. Second, incorporating the Facebook dataset allows for greater information diversity in the analysis of*

comments. Relying exclusively on Twitter as a data source would lead to results that are skewed towards Twitter platform practices and the political representativeness of its users. Research by Barbera and Rivero (2014) has shown that Twitter users who engage in political discussions tend to be male, reside in urban areas, and possess extreme ideological preferences [88]. Moreover, Twitter's character limit constrains discourse complexity, inhibits reflexivity, and tends to encourage uncivil behavior due to the impersonal nature of the platform [89]. By including data from Facebook, we can broaden our understanding of the phenomenon across multiple platforms. Furthermore, previous studies on polarization and hate speech have heavily relied on Twitter data alone [33, 90, 91]. Our research aims to expand the scope by extrapolating our findings to other platforms, enabling a more comprehensive examination of the phenomenon."

This information can be found on Pages 19-20, Lines 535-555.

Comment 3. Another conceptual issue, that spans into the methodology, is the adoption of an 'off-the-shelf' classifier for toxic discourse that is disconnected from broader discussions about online incivility and implies that 'toxic' = bad, perhaps leaning on an outdated view of the 'online public sphere' that is driven by a scarce literature review.

>>> Thank you for this comment. It echoes Reviewer 1's comment (Comment 1) about how "toxicity" is not necessarily detrimental to deliberative discourse. We agree with this point, and the text we added on Page 5, Lines 103-112, should address that general issue. We chose to use a standard classifier for "toxic" discourse in order to build from existing methodological tools, as the goal of the present paper is not methodological innovation. Rather, our goal is more of a data science and sociological analysis of the relationship between neutral bubble reachers' network positions and the styles of discourse they inspire. For this, linking comments on bubble reacher accounts to the standard classifiers is a way to incrementally build on past work in order to gain sociologically relevant insight about when and where neutral bubble reachers do or do not provoke "toxic" discourse – without, as noted above, presuming that such "toxic" commentary is necessarily detrimental to deliberation. Nevertheless, the question about the style of discussion inspired by those accounts situated between ideological partisans strikes us as an important question that has not yet been examined at this scale in the literature.

Comment 4. Still in the realm of conceptual and framing issues, the paper often refers to populist discourse to explain the results in Brazil, but that perspective is not grounded in any data and seems to suggest that the problem in the country is right-wing populism, whereas populism, as typically measured in surveys, is typically quite high in Brazil regardless of political leaning, being a weak explanatory concept to understand the underlying dynamics that the paper seems to unveil.

>>> We agree that populism in Brazil is not simply a right-wing phenomenon. Though we illustrate how populism, media hostility, and the online sphere intersected with Bolsonaro's presidency, our intention was not to argue that populism in Brazil is simply right-wing. Rather, our claim is that polarization and populism in general are more pronounced in Brazil compared to Canada. For data on polarization, we cite Orhan (2022) and Van Der Veen (2021). To further clarify, we included an additional citation to the YouGov-Cambridge Globalism Project, which also finds that populist attitudes are strongest in Brazil, while Canada ranks near the bottom among the countries surveyed. Finally, we now explicitly state that *"While populism has had a significant steady influence on Brazil's politics on both the left and the right since the early 20th century, its role in Canadian politics has been small by comparison."* (see page 9, lines 219-221). Since the dynamics we seek to explain are how neutral bubble reachers operate differently in contexts where polarization/populism are high vs. low, we believe that the comparison between the two countries is effective.

Comment 5. In terms of the methodology, (1) there is no clear rationale as to why you have datasets from different platforms that are no comparable across categories, e.g. with biased datasets coming from Facebook pages and the 'neutral' datasets coming from comments in pages. Moderation practices across platforms are quite different because even news outlets are limited in the ways they are able to moderate content on Facebook, whereas they typically employ heavier moderation strategies on their own websites. In the same vein, one explanation for some results in Canada has to do with a website using Facebook for comments, which is not comparable to native comments. The methods section seems to ignore such discrepancies and does not provide a convincing explanation for the approach.

>>> Thanks for this comment. We acknowledge that this is a limitation of our work, and because of that, we take some measures to address these concerns in Appendix S3 and S4. In S3, we show that toxicity scores are not products of the Facebook platform alone. Overall, variation in toxicity across studied pages suggests that toxicity is not a constant feature of the Facebook platform, i.e., it can have low and high toxicity for biased and neutral outlets; thus, we shouldn't expect our findings to be reducible to the influence of Facebook. S4 complements this sensitivity analysis. To ensure our results are not biased by platform selection, we consider comments

made in each news media on their Facebook account or website. S4 indicates that Facebook's influence on toxicity results is lower than expected.

Comment 6. (2) and related to the point about toxicity above, using off-the-shelf algorithms to classify text at scale is not sufficient to contribute to the understanding of online toxicity as a societal issue, as it does not advance our knowledge about context or role of toxic discourse online. Many have argued that online incivility may relate to 'deliberative' discourse traits, and research on perceptions of incivility demonstrates that individuals have a wide variation in the types of expressions that they see as uncivil. As such, a single measure of toxicity based on an algorithm that has not even been validated in the data it's been applied to is not particularly enlightening to learn about the dynamics underlying polarization and public discourse.

>>> Thanks for your comment. Our focus wasn't to create or validate a new toxicity measure but to study a broader phenomenon. We relied on a widely used machine learning model to generate the toxicity score, which was used as a proxy for uncivil/aggressive/conflictual discourse. To make it more apparent in the text, we inserted more references supporting the claim that Perspective API has been used in relevant recent studies in the literature to identify toxicity and also inserted the following discussion in the paper:

> "Previous studies indicate that Perspective API satisfactorily captures the toxicity of social media content [74–76]. For instance, Rajadesingan et al. [74] show that Perspective API's performance in identifying toxicity is comparable to toxicity labelled by humans. Some studies suggest that Perspective API has the potential for racial bias against speech performed by African Americans [95], but this possible bias should not compromise our analyses."

This information can be found on Page 23, Lines 613-618.

Comment 7 Importantly, polarization here seems to be related to a country characteristic: there are no measures of polarization and the biased datasets appear to be one-sided, mainly including right-wing accounts.

>>> Regarding the polarization measure for a country, we based this information on two previous studies available in the literature:

- Van Der Veen, O. (2021). Political Polarisation Compared: Creating a Comprehensive Index of Political Polarisation (Doctoral dissertation, Doctoral dissertation, Central European University).

- Orhan, Y. E. (2022). The relationship between affective polarization and democratic backsliding: comparative evidence. Democratization, 29(4), 714-735.

Both studies observe greater recent levels of affective polarization in Brazil as compared to Canada.

This information can be found on Page 9, Lines 226-228.

Regarding the biased datasets, BIASED_pt can be considered mostly right-wing. However, the accounts composing the dataset BR_BIASED_en, according to www.allsides.com/media-bias, are left-wing or lean toward the left. Please also note that both biased datasets present a similar toxicity level.

We inserted this information in the updated version of our manuscript in the "Bubble Reachers Comparative Datasets" Section.

Comment 8. All in all, I struggle to see a scientific advancement of our collective understanding of a) online toxicity and b) its relationship with polarization based on an analysis of an off-the-shelf classifier on a subset of accounts that supposed to be 'neutral' or biased based on other studies in other platforms. This study does not advance the understanding of news accounts and comments, nor toxicity classification, nor actual political polarization.

>>> Thanks for your comments. We believe the answers to the comments above help to clarify your concerns regarding our study.
