# Supplementary material for: Bubble reachers and uncivil discourse in polarized online public sphere
Source: PLoS One. 2024 Jun 20;19(6):e0304564. doi: 10.1371/journal.pone.0304564 (PMC11189196; doi:10.1371/journal.pone.0304564)
Supplement: Attachment — Submitted filename: responses.pdf [file pone.0304564.s008.pdf]

Bubble reachers and uncivil discourse in polarized online public sphere

Jordan Kobellarz, Milos Brocic, Daniel Silver and Thiago H. Silva

To the Editor and Reviewers.

We would like to thank you for reviewing our manuscript. We have revised the paper taking into account the Reviewers' remarks and suggestions, which were quite helpful. We believe that we have a much better version of the manuscript after incorporating those changes. Below, we present our responses to specific comments.

Responses to the second round of reviews

Reviewer #1

Comment 1: "I appreciate the opportunity to re-review the manuscript, and I appreciate the rigor and effort that the authors have put into the revisions. Basing my response on the initial comments that I provided, I believe that the authors have effectively addressed my concerns (as well as those raised by the other reviewers).

The only area where I would still encourage some minor revisions is the issue of better connecting these findings to the literature around confirmation bias and hostile media effects. The authors made some minor notes on this, but the connections remain a bit under-developed. I still believe that there is a rich opportunity here to better connect to this important area/body of literature. I would encourage the authors to consider an additional paragraph in the discussion addressing this. Can we make any inferences from these findings that might apply to the literature around confirmation bias? Does this study point to any novel opportunities for future research in that area?

Beyond that, I believe that my prior comments have been well-addressed."

>>> Thank you for these comments, and your suggestion to develop the connection to hostile media effects and confirmation bias. This is an important connection, and we point in that direction in the revised Discussion & Conclusion section. Specifically, we note that our study suggests that an individual's behavioural response to media outlets depends on how they are interpreted within a wider political context. In addition to partisan identity cueing individuals into hostile media perceptions and motivating their incivility, we suggest a populist context may cue hostile media perceptions more generally, motivating greater incivility across the board. While we point in this direction in the present study, the Conclusion suggests directions for developing these ideas further, such as examining variation in responses to media outlets across more contexts and over time.

Reviewer #3

Comment 1: "The authors present a complex study with several distinct data sets aimed at testing the relationship between polarization on the national level and toxicity in the comment sections of two different types of communicators: large scale rather neutral news outlets as compared to more partisan outlets. Albeit I do think that there is clearly some merit in the paper, there are several aspects that prevent me from recommending its publication in the current state. I know that this is surely not the result the authors had hoped for, moreover as this a revised manuscript and I am new on the board. I will thus refrain from reiterating the comments made by the other reviewers before. They are all legit and I am not sure if they were addressed all sufficiently, but it is an editorial task to judge this. Instead, I will provide some additional nuances (albeit they do reiterate the sentiment from the already existing reviews). I hope that they will find my comments useful in polishing their work.

(1) Theoretical rigor. Albeit I do understand the disciplinary origins of the term bubble reacher, I do not think that it does a good job in linking the study to the existing research out there and I am not convinced of the implications of the metaphor. I have two reasons for my concern: (a) the "neutral bubble reachers" seem to be basically legacy news sources. So why not call them like that? This would allow tons of social sciences to relate to the findings as they potentially tell us larger stories about public spheres (something communication science is interested in), the normative ideal of deliberation vs. conflicts (something political philosophy will be interested in),

or the role of news in polarized contexts (which would be of interest to political science amongst others).

Through the analogy of the bubble reacher, the (pretty much debunked) concept of the filter bubble is reiterated despite exactly these accounts contradicting the original filter bubble hypotheses. And there is no evidence that fragmentation itself does foster toxic discourse (p.5). This strongly depends on the communication norms in the disparate communities (Gibson, 2019).

Also, readers will find it likely easier to understand what legacy news vs. partisan news are, while bubble reacher is a new term they need to recall over and over again. Furthermore, to convince me of the term, I would like to see the generalizability of findings to other "bubble reachers" out there such as completely unpolitical accounts. Do the hypotheses hold for Taylor Swift too? This question seems to be simple, but the authors provide the (interesting) idea that "[bubble reachers] offer spaces that mitigate rancorous discourse by providing a common starting point for conversation, even if that starting point is differently interpreted? Or do they provide the discursive equivalent of "combat zones," gladiatorial arenas where partisans meet not to deliberate and converse, but to fight?" – This is basically the idea of either deliberative or confrontative theories about the public sphere (Habermas, 2006; Mouffe, 2018). Thinking through these questions and a precise wording will help future scholars to built upon this work.

This might also help to remove a simple alternative hypothesis that could explain the data equally well: All differences in the comment sections are due to differences in moderation and not the "attraction of toxicity". In polarized countries, legacy news try to cater to a more split audience and thus moderate less (I am not saying that this is the case, but there is nothing in the theoretical argument or the paper that does say otherwise)

>>> Thank you for this comment. We appreciate the reviewer's reservations about the term "bubble reacher," but we have decided to retain it in the revision. Specifically, the term "bubble reacher" arises out of existing literatures in computer and network science, and as this is the origin of our own study, we feel that the concept fits well in this discursive context and is legible to that audience. We note this on Page 6, Line 123. Nevertheless, we have sought to incorporate key intuitions in the comment. We followed the reviewer's advice to clarify the nature of fragmentation online, noting that the issue is not primarily about epistemic closure, and a lack of exposure to outside information, but rather epistemic discrediting, where outsider information is readily dismissed (see Page 5 and 6, Lines 108-122). We also noted the

importance of communication norms, and cited Gibson (2019). While clarifying the nature of fragmentation online, we believe our question on whether those meeting places that bubble reachers afford are sites of greater or lesser incivility remains pertinent.

At the same time, we take seriously the reviewer's point about the intuitive nature of references to legacy vs. partisan news outlets. To highlight this connection, in the revision, we note that neutral bubble reachers are largely legacy news outlets. See Page 6, Lines 142-144. The revision also highlights that the bubble reacher metrics do not pre-determine which outlets they would identify; rather legacy outlets are empirically more likely to be identified as neutral bubble reachers (see Page 7, Lines 154-156). The bubble reacher concepts and metrics are analytical. Still, the fact that they pick out legacy vs partisan media is important and helps to give them intuitive meaning, which we hope will make the analysis interpretable by additional audiences. Thank you for pushing us to include and clarify this.

The reviewer raises two other important points in this comment. The first concerns non-political accounts. While fully exploring this issue would take us well beyond the scope of this study and require a different paper, we note that we did examine the extent to which incivility spills over to non-political discourse in Brazil (see Pages 26-29, Lines 706-778). Still, we acknowledge this could be pursued further and note that in the conclusion (see Page 33, Lines 915-920). Secondly, the reviewer wonders whether moderation practices drive the outcomes. This too is an important question. Our supplementary analyses addressed this as far as we could with available data, by comparing results between an outlet's comments when they are moderated in-house vs. on Facebook. We do this by analyzing the correlation between comments toxicity made on the website and those on the Facebook page separately for Yahoo News and the New York Times cases (see Pages 43-44, Lines 1110-1132). We recognize this is not a perfect test, and note this as a limitation and opportunity for additional research (see Page 44, Lines 1133-1139 on Appendix 6).

Comment 2: Somewhat related is the (computer science based) use of toxicity. I would encourage the authors to engage with the theoretically more nuanced concept of incivility instead as it seems to be a better fit to their overall framework (Coe et al., 2014; Muddiman & Stroud, 2017; Ziegele et al., 2014)"

>>> Incivility does seem to more closely align with the theoretical orientation of our study, and so we have taken this helpful suggestion to heart to feature the notion of civility/incivility more centrally, both in the literature review and in the discussion and conclusion (see Page 5 Lines 97-107, Page 10 Lines 253-258, Pages 30-31 Lines 820-835). Thank you for this point.

This also led us to revise the way we discuss empirical metrics we employ, so that we now consider the toxicity score as an empirical proxy for incivility. We discuss this approach in the text on Page 10, Lines 260-278. Concretely, this meant we revised sentences to include the term “incivility” where appropriate and maintained the term “toxicity” only where it referred specifically to the measure generated algorithmically (the “toxicity score”). Also, to better relate these concepts, we added the following paragraph in the section “Detecting Uncivil Discourse in Canada and Brazil”:

> *“While the perception of incivility in discourse is to some degree a subjective matter, the research literature identifies some characteristic features that demarcate incivility, such as ad hominem attacks (direct attacks on the person instead of their arguments), vulgarity and exaggerations [64]. In short, incivility involves communication that violates norms of courtesy and respect in social interactions, especially in the political sphere, where discussion of ideas and opinions can be heated. In the online sphere, incivility theoretically overlaps with “toxic discourse,” which refers to disrespectful or irrational comments that may lead users to leave discussions [65].”*

This information can be found on Page 10, Lines 256-260.

Comment 3: (2) Methodological adequacy. Overall, the methods seem to be sound. The comparative context is well chosen and justified. The composition of the data sets was really hard to understand, and abbreviations do seldomly make anything better in terms of readability. The study seems to realize a 2 (Canada vs. Brasil) × 2(legacy vs. partisan news) design that is complemented by different robustness checks in the supplementary material. But it took me a separate paper to understand that. This should not be the case. A simple cross table could help to visualize and describe these four main data sets.

>>> Thank you for the suggestion to include a cross table summarizing the main datasets. We've now incorporated a 2x2 table that follows a similar approach. This table features two rows representing media types ("Neutral Bubble Reacher" and "Partisan") and two columns

representing national polarization levels ("High" and "Low"). We believe this table effectively summarizes our main datasets comparisons. This table can be found on Page 23.

Regarding the dataset abbreviations, we've made updates to a non-abbreviated version and renamed some to align with the changes suggested in the comments above. The table below illustrates the transition from the old names to the new ones:

**COMPARATIVE DATASETS**	
**OLD NAME**	**NEW NAME**
BR_NEUTRAL_pt	NEUTRAL_REACHER_pt
BR_NEUTRAL_en	NEUTRAL_REACHER_en
BIASED_pt	PARTISAN_pt
BR_BIASED_en	PARTISAN_REACHER_en
**REFERENCE (BASELINE) DATASETS**	
**OLD NAME**	**NEW NAME**
TOXIC_en	UNCIVIL_en
TOXIC_pt	UNCIVIL_pt
NEUTRAL_en	CIVIL_en
NEUTRAL_pt	CIVIL_pt
**SENSITIVITY ANALYSIS DATASETS**	
**OLD NAME**	**NEW NAME**
FB_NEUTRAL en	FACEBOOK_NEUTRAL_en
FB_BIASED_en	FACEBOOK_PARTISAN_en
FB_PERSON_en	FACEBOOK_PERSON_en
FB_OTHER_en	FACEBOOK_OTHER_en
NYT_SITE_en	No change
YAHOO_SITE_en	No change
G1_SITE_pt	No change

Comment 4: I also completely lost track when all the other Facebook data sets came into play, and I would encourage the authors to move all (laudable) additional analyses that are not directly relevant to testing the hypotheses in the supplementary material. This would also reduce the readers confusion about all the different platforms used to get the data for the "baseline" comparisons (another term I found highly misleading as the one data set seems to be the toxic extreme rather than the baseline). Furthermore, the actual number of posts in each of the datasets should be clearly visible in that table.

>>> We moved all additional datasets and sensitivity analyses unrelated to H1, H2, H3, and H4 to the supplementary materials in Appendix 1 to 6. As a result, the methodology and results are now focused on the main analyses, which are exclusively related to the hypotheses H1, H2, H3 and H4. We also replaced the term "baseline" with "reference," which is more appropriate for referring to datasets with extreme cases to compare with other datasets. Finally, we included the number of comments in each dataset in the 2 x 2 table presented in the previous comment.

Comment 5: (3) Analytical Rigor. The labeling of the central variable of interest was done algorithmically. As such the authors should manually validate the suitability of the algorithm to detect toxicity and report the results of these robustness checks. This is particularly relevant given the reported (!) bias against non-standard English (p.23) and the intercultural comparison.

>>> Thanks for your comment. We relied on a widely used state-of-the art multilingual model to generate the toxicity score, which was used as a proxy for uncivil discourse. This model has been developed and scrutinized by scholars over the last 5 years since it was made available to the general public. Recently, state-of-the-art results were presented in a study (Lees et al., 2022) that systematically tested known vulnerabilities in toxicity detection, by comparing state-of-the art multilingual models, showing that the latest available Perspective API production model outperforms strong baselines (the same used in our research).

Due to the complexity involved in testing a Large Language Model like the Perspective API using standard methods, along with the findings from studies that assessed its robustness across various contexts using well-established datasets for diverse tasks and addressing multiple vulnerabilities, we opted to rely on these existing assessments rather than conducting additional robustness tests. Undertaking such tests would significantly complicate this research

and detract from our primary objective of examining the impact of bubble reachers on incivility as compared to partisan accounts in distinct political cultures.

To clarify this in the article, new references were added to support the robustness of the chosen method for toxicity detection:

> "Given that this is a widely adopted tool by major news outlets for moderating comments on their portals, in addition to the fact that it is openly available, having support and good performance on different languages [79], Perspective API was chosen to be applied to this research. Previous studies indicate that Perspective API satisfactorily captures the toxicity of social media content [81–83]. For instance, Rajadesingan et al. (2020) [81] show that its performance in identifying toxicity is comparable to toxicity labelled by humans. Some studies suggest that Perspective API has the potential for racial bias against speech performed by African Americans [97], but this possible bias should not compromise our analyses. A recent study systematically tested for adversarial examples and other recognized vulnerabilities in state-of-the-art multilingual models for toxicity detection and found that the latest production model of the Perspective API, the one used in this study, outperforms strong baselines [76]. Considering the widespread adoption of this tool and its state-of-the-art performance, we deem this tool suitable for use in this research."

This information can be found on Pages 20-21, Lines 562-576.

Comment 6: The preprocessing was done carefully but should be reported in the supplementary section to.

>>> As suggested, we have moved the comment preprocessing step to Appendix 3.

Comment 7: I am less convinced of the chosen statistical approach. First, the authors should provide a table with the means and standard deviations for toxicity in each context.

>>> Thanks for the comment. In the updated version of the manuscript, we included the mean, median and standard deviation values for each dataset and respective sources in Appendix 2.

Comment 8: I would also encourage them to think about the smallest effect size of interest (SEIO) for their paper (Lakens, 2017). Their argumentation is very often "yes, there is a difference, but the difference is small" (e.g., p. 27). But small is not necessarily irrelevant, particularly when we talk about news that potentially get millions of views.

>>> We appreciate these incisive comments, which make us reflect on possible ways to make the results as robust as possible; thanks for that. The two one-sided tests (TOST) procedure for equivalence tests discussed by Lakens, 2017 is not an appropriate choice for our data because: i- normality, a necessary condition, is not presented in our datasets; ii- there is uncertainty in how to define a range to be considered the smallest effect size of interest that makes sense in our context – this is a challenge of the TOST strategy and, despite having some heuristics for that, they are mostly recommended for the health domain; iii- we are less interested in possible effects in specific ranges, instead it is more important in our case to identify general kinds of differences in distributions than investigating whether the difference between these groups is zero based on certain locality measures, such as mean, or intervals around them; iv- our data scale is much larger than typical conditions where this procedure is demanded, where nonsignificant test results are hardly indicative of the absence of a true effect. In order to improve our manuscript, especially in parts where we argue about the size differences, we revised the method used to compare distributions. Instead of analyzing by groups, we are now using a method to enable pair comparisons – the Kolmogorov–Smirnov test. Nothing in the conclusions changes, but this provides us with a better tool for specific cases where we point out comparisons regarding differences. The updated version of the manuscript was updated to reflect these changes.

Comment 9: After that, they should turn to the main analyses. Here the authors actually had a 2 × 2 design and should test it as such, discussing the main and interaction effects followed by pairwise comparisons (this would also allow them to say: "neutral" vs. "partisan" news have an effect but that is moderated by context, which seems to be what they are interested in).

>>> Following your suggestion, we created a new 2 x 2 table to summarize results, keeping the same structure used in the 2 x 2 table created to summarize the main datasets for H1, H2, and H3 presented in comment 3. This table can be found on Page 26. Thank you for the helpful idea.

Comment 10: Second, a simple statement that Kruskal-Wallis tests were used to account for the (non-normal) data structure. Then they should report test after test and clearly state if that result was consistent with their hypotheses or not. Currently, the result section is very long and hard to follow.

>>> Thanks for the comment. As we mentioned in reply to comment 8, we replaced the Kruskal-Wallis test with the Kolmogorov–Smirnov test. Now we can provide comparisons test by test (pairwise) instead of heaving a result by a group. We agree with the reviewer that providing this information to the reader is important. Thus, to make the results less challenging to follow, we opted to provide these test results in an external file (https://drive.google.com/file/d/10PvfRN2gbFGGrlbLwgzdODHdyipGwsY2/view?usp=drive_link) – we could not find a good way to append this information to the text without increasing the complexity of the information already provided. References to this file have been included wherever such data is needed.

We also provide the pre-processed dataset and a repository with all the necessary code to ensure the replicability of the results. The dataset can be found at https://drive.google.com/file/d/1ihiYa9_u_HfXOaNP6qWHfbAV80z6XeRO/view?usp=sharing and the repository with the code at https://github.com/jordankobellarz/bubble-reachers-incivility. Links to all these materials were included on the research website (https://sites.google.com/view/onlinepolarization). Therefore, we included this information in Page 4, Lines 88-90:

> *"The pre-processed dataset and all the code necessary to replicate the statistical tests carried out in this research were made publicly available on the project website: https://sites.google.com/view/onlinepolarization"*

Comment 11: (4) Minor issues:

• p.1, l. 13 - the authors might want to quote some of the early optimistic scholars believing that the internet would be a "school for deliberative democracy"

>>> Good point. We softened the claim to read now that the internet "could" be a school for deliberative democracy and included two citations:

Dahlberg, Lincoln. 2001. “The Internet and Democratic Discourse: Exploring The Prospects of Online Deliberative Forums Extending the Public Sphere.” Information, Communication & Society 4(4):615–33. doi: 10.1080/13691180110097030.

Price, Vincent. 2009. “Citizens Deliberating Online: Theory and Some Evidence.” Pp. 37–58 in Online Deliberation: Design, Research, and Practice, ed. Todd Davies. Chicago, IL: University of Chicago Press.

• P.2, l. 31 given the numeric referencing system, I would recommend reformulating the sentence and actually name the authors here (as done in the following sentence)

>>> We updated the sentence, including the names of the authors when citing them for the first time:

“To examine these dynamics, we extend Kobellarz et al. (2022) [16], which investigated the role of central Twitter users, whom they characterized as “bubble reachers.” Bubble reachers are users who distribute content that reaches other users with diverse political opinions [16]. In order to identify these central users, the authors created a novel centrality metric called intergroup bridging [16].”

This information can be found on Page 3, Lines 31-35.

• P. 3, l. 70 surely a question of style but the hypotheses come pretty out of the blue and are not derived from the text that comes before them. That reduces their convincing potential.

>>> Considering that the summarized hypotheses and results could reduce the convincing potential of the text, we considered it appropriate to remove them from this section.

• P. 3, l. 78 - I also find it unusual to read the results before the actually paper (but that might be an interdisciplinary issue). Personally, I would delete the abstracts starting l.65 and l.78 and continue directly with the explanation of the papers structure

>>> We agree that presenting the hypotheses in this section, without having presented the theoretical background, makes it less convincing. Therefore, we removed the hypotheses, as well as the results summary from this section, resulting in a more concise paragraph:

> *“Addressing these issues is the central goal of this paper: we examine how ideologically neutral bubble reachers differ from ideologically partisan accounts in the level of uncivil discourse they provoke, and explore how this varies in a context where populism is more and less prominent in political culture. We do that by examining the contexts of Brazil, where populism has deep roots in the political culture, and Canada, where the political culture is comparatively moderate. By analyzing the communication style provoked by neutral bubble reachers and ideologically partisan accounts in these distinct political cultures, this study marks a substantial step forward regarding past research.”*

This information can be found on Page 4, Lines 66-74.

• Figures should be readably for people who are color blind

>>> We update the figures using the Okabe-Ito colour palette (Okabe & Ito, 2008), which is accessible to people with a variety of forms of colour blindness.

Reference:

Ichihara, Y. G., Okabe, M., Iga, K., Tanaka, Y., Musha, K., & Ito, K. (2008, January). Color universal design: the selection of four easily distinguishable colors for all color vision types. In Color Imaging XIII: Processing, Hardcopy, and Applications (Vol. 6807, pp. 206-213). SPIE.

• Is it correct that the comments in Brasil legacy news were more toxic than the comparative data set with very toxic Wikipedia comments? (Figure 1)

>>> The Wikipedia dataset (UNCIVIL_en) presented an average toxicity of 0.96 with a standard deviation of 0.06, while the Brazilian legacy news dataset (NEUTRAL_REACHER_pt) presented an average toxicity of 0.42 with a standard deviation of 0.26. Therefore, it is safe to say that NEUTRAL_REACHER_pt is less toxic than UNCIVIL_en. In fact, in Figure 2, the boxplots indicate that the UNCIVIL_en dataset is the most toxic among all.

Comment 13: References

Coe, K., Kenski, K., & Rains, S. A. (2014). Online and uncivil? Patterns and determinants of incivility in newspaper website comments. Journal of Communication, 64(4), 658–679. https://doi.org/10/f6dxrx

Gibson, A. (2019). Free speech and safe spaces: How moderation policies shape online discussion spaces. Social Media + Society, 5(1), 205630511983258. https://doi.org/10.1177/2056305119832588

Habermas, J. (2006). Political communication in media society: Does democracy still enjoy an epistemic dimension? The impact of normative theory on empirical research. Communication Theory, 16, 411–426. https://doi.org/10.1111/j.1468-2885.2006.00280.x

Lakens, D. (2017). Equivalence tests: A practical primer for t tests, correlations, and meta-analyses. Social Psychological and Personality Science, 8(4), 355–362. https://doi.org/10/gbf8nt

Mouffe, C. (2018). For a left populism. Verso Books.

Muddiman, A., & Stroud, N. J. (2017). News values, cognitive biases, and partisan incivility in comment sections. Journal of Communication, 67(4), 586–609. https://doi.org/10/gbr4c8

Ziegele, M., Breiner, T., & Quiring, O. (2014). What creates interactivity in online news discussions? An exploratory analysis of discussion factors in user comments on news items. Journal of Communication, 64(6), 1111–1138. https://doi.org/10.1111/jcom.12123
