# Supplementary material for: Bubble reachers and uncivil discourse in polarized online public sphere
Source: PLoS One. 2024 Jun 20;19(6):e0304564. doi: 10.1371/journal.pone.0304564 (PMC11189196; doi:10.1371/journal.pone.0304564)
Supplement: Attachment — Submitted filename: Response to Reviewers.pdf [file pone.0304564.s009.pdf]

Bubble reachers and uncivil discourse in polarized online public sphere

Jordan Kobellarz, Milos Brocic, Daniel Silver and Thiago H. Silva

To the Editor and Reviewers.

We would like to thank you for reviewing our manuscript. We have revised the paper taking into account the Reviewers' remarks and suggestions, which were quite helpful. We believe that we have a much better version of the manuscript after incorporating those changes. Below, we present our responses to specific comments.

Responses to the third round of reviews

Reviewer #3

Comment 1: "I want to congratulate the authors to their revision. Although I still think that we don't need more papers that contribute to the already persistent myth of filter bubbles, I acknowledge the authors desire to stick with the metaphor due to the usual language in their discipline. I think that the paper has substantially improved and is now much better grounded in the literature and I think that it will be of great interest to the audience of PLOS One."

>>> Thank you for your feedback and acknowledgment of the improvements made to the article. We're glad to hear that the paper is now better grounded in the literature and will be of significant interest to PLOS One's audience.

Reviewer #4

Comment 1: "There is a lot to like about this paper. I would like to particularly highlight the authors' profound knowledge of the literature. Different from many other papers on echo chambers and related issues, the authors show a nuanced, differentiated, realistic view on echo-chamber like phenomena and their effects. Altogether, this paper seems to me like the result of a truly successful interdisciplinary collaboration. The methodological design is well-developed and justified and explained in a well-understandable way. I have some comments on how to further improve the paper, but I want to point out that these are not fundamental in nature and can relatively easily be fixed."

>>> Thank you for your careful review and guidance. Below, we present our considerations for each of the comments provided.

Comment 2: "The authors seem to have a clear understanding of echo chambers and filter bubbles and the differences between both, which becomes apparent several times in the paper when they implicitly refer to that. However, I think the paper would benefit if the authors clearly defined both terms and explained the difference between these phenomena already in the introduction."

>>> An explanation of these concepts was added on Pages 2-3, Lines 22-30:

> *"The metaphor of "echo chambers" [12] refers to epistemic environments in which individuals or groups interact with like-minded people or sources that reinforce their existing beliefs or opinions. In an echo chamber, information, ideas, and opinions are "echoed" and "amplified" within a closed network, leading to a reinforcement of existing viewpoints and a lack of exposure to diverse perspectives [12]. While the concept of "filter bubbles" refers to the algorithmic filtering that selectively curates content based on a user's past behavior, preferences, and demographic information [38]. In a filter bubble, users are presented with information that aligns with their interests and beliefs while filtering out opposing viewpoints or dissenting opinions [38]."*

Comment 3: "The same applies to populism which is a quite central concept in this study but the paper lacks a definition and conceptualization of it. Please define what you mean with populism and populist communication. And be aware that all political actors make use of populist communication, not only so-called populists (e.g., Ernst et al., 2019; Lilleker & Balaban, 2021). In the same vein, I wonder if H3 and H4 should rather be about contexts that are MORE populist and polarized. As they read now, it sounds like there was a binary distinction between populist/polarized on the one hand and non-populist/non-polarized contexts on the other hand. However, both populism and polarization are rather continuums on which countries can be more or less populist/polarized. Regarding the example of populism, it is worth mentioning that anti-elitism and being rather exclusive is typical for populism in Latin America, different from the US and Europe (Rovira Kaltwasser, 2012). It would also be a good idea, I think, to mention that anti-elitism is often typical for left-wing populism while right-wing populism is often rather characterized by excluding outgroups (Reinemann et al., 2017)."

>>> Thank you for the suggestion. We have now clarified how we understand populism as a discursive style that can be directed to many goals and ideological aims (Page 8, Lines 193-210). Likewise, we agree with your suggestion to modify our hypotheses to make it a question of more or less populism/polarization, rather than treat these differences as a binary (Page 9, Lines 237-240).

Comment 4: "Rightly, the authors point out that bubbles or echo chambers let in contrary content but it is inside the bubble not presented in a neutral way but rather "filtered through a partisan lense" (lines 62-63). I would like to recommend a paper by Bright et al. (2020) to the authors which may help to further strengthen this argument."

>>> Thank you for recommending this paper. It complements our arguments quite well. We've now incorporated the paper in our literature review on page 6, Line 124-126:

> *"According to Bright et al. (2020), echo chambers thrive on steady exposure to low levels of oppositional views -- they rely on conflict to enliven group identity, while providing cognitive tools to manage dissonance in ways that reinforce extreme belief."*

Comment 5: “The term “platform affordances“ is a quite vague one with many different meanings and understandings. Therefore, I recommend avoiding this term and replacing it by something different (e.g., platform characteristics).”

>>> Thanks for pointing that out. We agree that the term "affordances" is inappropriate for the context where it was cited, so we replaced the two occurrences in the article with "characteristics."

Comment 6: “Unfortunately, I don’t understand the following sentence: “As Alexander [47] observes, populists portray claims to impartial expertise by mediating institutions as a cover for narrower interests of the status quo.“ Please make clearer what you mean with this.”

>>> Thanks for the suggestion. We have revised the sentence, and hope it is clearer now (Page 8, Lines 200-203):

> *“As Alexander (2020) observes, a common trope in many populist scripts involves the critique of impartial expertise, in which experts’ claims to neutrality are reinterpreted as partisan defenses of “establishment” interests that run counter to those of the “common people.”*

Comment 7: “I agree that Brazil and Canada are good cases for the comparison in this paper. However, the country selection would be even more convincing if the authors could give some more information about structural similarities/differences between both countries: Is this a most similar systems design with the different degrees of populism and polarization being the only probable reasons for the differences found? Or might there be alternative structural explanations? This should also include some numbers on the use of Facebook and other relevant media.”

>>> Thanks for this suggestion. We’ve strengthened our case selection by noting certain similarities in structure, and online engagement between Brazil and Canada, despite differences in levels of populism and polarization on (page 10, lines 266-278). However, we also reiterate that extending our analysis to incorporate more cases beyond Canada and Brazil could be a worthwhile direction for future research (page 34, lines 940-944).

Comment 8: "Regarding the literature review, the authors show in-depth knowledge on computational studies in their field, but I think it would be good to add some manual content analyses on incivility in user comments."

>>> Thank you for the recommendation. We have incorporated a citation to a manual examination of comments sourced from one of the datasets utilized in this article (NEUTRAL_REACHER_pt) (Kobellarz & Silva, 2022). Additionally, we provide examples of both civil and uncivil comments. See pages 11-12, lines 239-302:

> *"To provide context, these are examples of web comments obtained from one of the datasets studied in this article, which were manually annotated by three independent evaluators regarding their toxicity [69], demonstrating uncivil discourse: "tirso, you drunk, I'm not just a man on the internet, come here or give me your address if you're a man, and I'll come and talk to you in person. My address is [...], (it's very close to the current presidency of your luladrão boss) come here and talk this nonsense" and "KKKKKK now DataFAKE does a minimally truthful survey, because it was already getting bad. kkkkkkkkk ridiculous! They tried to manipulate the Brazilian vote but this is over!! they just lost the rest of the credibility they had, if they had any!". Meanwhile, these are examples of comments also manually annotated demonstrating civilized discourse [69]: "In the debate for governor of Rio held on Globo on the 25th, Eduardo Paes scored a great victory, he deserves to be elected." and "Bolsonaro did not claim medical recommendation, the doctors themselves gave an interview recommending that he not go. Everyone saw it."*

Comment 9: "The dataset PARTISAN_pt, as I understand, only includes conservative Facebook pages. Is it a problem that it does not contain any left-wing pages? Regarding CIVIL_pt and CIVIL_en, I wonder why the authors did not use equivalent websites in both countries and how this might affect the cross-country comparability of their data."

>>> For the PARTISAN_pt case, we complemented the limitations section with (page 34, lines 916-922):

> *"Additionally, it is noteworthy that PARTISAN_pt exclusively comprises right-wing pages, whereas PARTISAN_REACHER_en includes left-wing or left-leaning pages, as indicated by the All Sides Media Bias Report. This distinction is pertinent, as varying political perspectives*

may prompt disparate online behaviors in terms of information consumption [96] (e.g. liberals being more likely than conservatives to engage in cross-ideological dissemination) [39]).".

For the case of CIVIL_pt and CIVIL_en possibly not being equivalent (and other similar cases, where distinct platforms were included as equivalents), we included in the limitation section a passage that shows how it might affect the results (pages 33-34, lines 905-914):

"The fact that the comparative, reference and alternative datasets were obtained from different sources makes comparisons less robust, since the dynamics of interactions on each platform can influence the degree of incivility. For example, NEUTRAL_REACHER_pt and NEUTRAL_REACHER_en comments were extracted from content produced on distinct news websites, while PARTISAN_pt and PARTISAN_REACHER_en comments were extracted from content on Facebook Pages in distinct situations and dates. In this sense, each source (news website) has distinct moderation mechanisms that may or may not allow uncivil behaviour in comments, thus making some news outlets less uncivil by default. These differences make it difficult to conduct a perfectly fair comparison between them. (...)"

Furthermore, the CIVIL_pt and CIVIL_en cases were specifically chosen because they had the lowest average toxicity compared to other datasets, which was a desirable characteristic for good base cases, as can be seen in Table 9 in Appendix S2. As these cases were used strictly as references, as well as UNCIVIL_pt and UNCIVIL_en, even if there is difference between platforms, this does not affect the result.

Comment 10: "For the examples given in the text which are in Portuguese, the authors should give English translations so that all readers can understand them."

>>> Thanks for the suggestion. In the updated version, translations were provided instead of the original texts.

Comment 11: "When it comes to the period under investigation, I wonder if it might have influenced the findings that the data were collected in the midth of the Covid-19 pandemic which polarized the public discourse in many countries. This is of course a hypothetical question but I think it is worth addressing it, for example in the discussion: Is it conceivable that the findings would have been different in a more routine phase?"

>>> Thanks for the comment. Most of our datasets do not cover strict periods due to COVID-19 in the countries studied – the majority are before the pandemic, but there is one during (G1_SITE_pt), one after (CIVIL_en), and another undefined (UNCIVIL_pt). However, as COVID-19 could have strengthened polarization in Canada and Brazil after the pandemic, we included this future step on Page 35, Lines 945-958:

> *"Further, examining the hypotheses in different periods for the same country is also an important future step; this enables the understanding of, for example, how changes in political culture affect the results. For instance, we explain higher incivility levels in Brazil due to greater polarization and populism in their political culture – evidence of longitudinal growth in incivility corresponding with polarization would be in line with this explanation and could parse out the time-ordering between these two. Likewise, a longitudinal analysis could examine how the diffusion of populist framing in public culture affects whether citizens adopt more toxic styles in their expression [124]. Another motivation for examining our hypothesis in a different period is because COVID-19 has been associated with an increase in affective polarization, exacerbated by factors such as the politicization of public health measures and information, leading to heightened ideological divides [125]. Although most of our data do not cover this period (only G1_SITE_pt and possibly UNCIVIL_pt, which does not provide their collection date), polarization could have changed after this period."*

Comment 12: "When it comes to news media as bubble reachers, the authors' argument could be strengthened by a study by Magin et al. (2021) which shows that news media helps people with more extreme opinions to maintain the connection with the common core of the public discourse."

>>> Thanks for pointing this reference out. We incorporated it in our manuscript. We also complemented the discussion by adding this text on Page 7, Lines 156-158:

> *"For instance, Magin et al. show that legacy media outlets help politically extreme individuals sustain engagement with the fundamental essence of public dialogue."*

Comment 13: "Regarding the findings, I wonder if differences between different news media types might help explaining them, e.g. the difference between quality newspapers and tabloids

or the difference between legacy media and alternative media. Maybe the authors could discuss that in the conclusion."

>>> Thanks for the comment. Given the overlap between ideologically neutral bubble reachers and legacy media, we agree that this may partially explain the findings we observed. However, as we note, this correspondence emerged out of our data, with our earlier work on detecting ideologically neutral bubble reachers finding that they tended to be legacy media outlets (Page 21, Line 569-570). As such, we primarily attribute our findings to the neutral bubble reaching quality of these outlets, as this helps unpack what is distinctive about the relational configuration of legacy media outlets. On page 32, lines 870-871 we elaborate how our findings speak to literature on legacy media. However, we note that our analysis is partially limited because of this overlap, and suggest that examining how these findings apply to bubble reachers that aren't legacy media outlets could be a worthwhile direction for future research on page 35, Lines 958-961.

Comment 14: "The explanation why the authors use one dataset including US media is first given in the discussion. This should be moved to the methods section."

>>> We rephrased and moved the explanation to the methods section (Page 16, Lines 449-456):

> *"For the English case, we cross-referenced the top 100 bubble reachers with partisan RP(H) in Canada with a Facebook dataset containing comments on 20,000 posts made by American news organizations and personalities [88]. This dataset has been labelled as PARTISAN_REACHER_en. The American political culture is recognized for its polarization [109, 110], and some evidence shows that it is higher than the Canadian scenario [59]. Despite differences in political culture, Canadians also engage with these sources, albeit to varying degrees. Thus, we selected this dataset as an approximation for partisan accounts for the Canadian situation."*

Comment 15: "Line 764: "uncivil" must be "uncivility".

>>> Thanks for the comment. This mistake was corrected in the updated version.

Comment 16: “References

Bright, J., N. Marchal, B. Ganesh, and S. Rudinac. 2020. Echo Chambers Exist! (But They’re Full of Opposing Views). doi: https://arxiv.org/abs/2001.11461

Ernst, N., Esser, F., Blassnig, S., & Engesser, S. (2019). Favorable opportunity structures for populist communication: Comparing different types of politicians and issues in social media, television and the press. The International Journal of Press/Politics, 24(2), 165–188. https://doi.org/10.1177/1940161218819430

Lilleker, D. G., & Balaban, D. (2021). Populism on Facebook. In J. Haßler, M. Magin, U. Rußmann, & V. Fenoll (Eds.), Campaigning on Facebook in the 2019 European parliament election: Informing, interacting with, and mobilising voters (pp. 267–282). Palgrave Macmillan. https://doi.org/10.1007/978-3-030-73851-8_17

Magin, M., Geiß, S., Stark, B. & Jürgens, P. (2022). Common Core in Danger? Personalized Information and the Fragmentation of the Public Agenda. The International Journal of Press/Politics 17(4), 887-909. https://doi.org/10.1177/19401612211026595

Reinemann, C., Aalberg, T., Esser, F., Strömbäck, J., & de Vreese, C. (2017). Populist political communication: Toward a model of its causes, forms, and effects. In T. Aalberg, F. Esser, C. Reinemann, J. Strömbäck, & C. de Vreese (Eds.), Populist political communication in Europe (pp. 12–25). Routledge. https://doi.org/10.4324/9781315623016

Rovira Kaltwasser, C. R. (2012). The ambivalence of populism: Threat and corrective for democracy. Democratization, 19(2), 184–208. https://doi.org/10.1080/13510347.2011.572619“
