# Supplementary material for: Bubble reachers and uncivil discourse in polarized online public sphere
Source: PLoS One. 2024 Jun 20;19(6):e0304564. doi: 10.1371/journal.pone.0304564 (PMC11189196; doi:10.1371/journal.pone.0304564)
Supplement: Attachment — Submitted filename: Response to Reviewers.pdf [file pone.0304564.s010.pdf]

Bubble reachers and uncivil discourse in polarized online public sphere

Jordan Kobellarz, Milos Brocic, Daniel Silver and Thiago H. Silva

To the Editor and Reviewers.

We would like to thank you for reviewing our manuscript. We have revised the paper taking into account the Editor final remarks.

Responses

Editor

Comment: Before we can proceed, we noted during editorial checks that there remain some instances of (censored) offensive language in your manuscript text, specifically on lines 770-777 of the current 'clean' manuscript file. We apologize that these were not identified and brought to your attention to address sooner.

While the Academic Editor does not object to the use of these quoted examples, in order to meet the journal editorial requirements, as previously discussed, we ask you to remove these quoted examples from your manuscript text. We otherwise appreciate your efforts to make the full uncensored dataset separately available.

>>> Thank you for bringing these instances to our attention. As per your request, we have removed the examples.

Reviewer #1

Comment: I have re-reviewed the manuscript twice now, and I feel that the authors have addressed all of my concerns (and those of other reviewers) excellently. The paper makes a strong contribution and I'm happy to recommend it for publication.

>>> We greatly appreciate the time and effort you have dedicated to this review process.

Reviewer #4

Comment: I would like to thank the authors for considering and implementing my comments in a convincing manner. I think the manuscript has further improved in quality and is now ready for publication.

>>> Thanks for your guidance, we believe the article has undergone significant improvement and is now well-prepared for publication.
